# Early feeding practices and eating behaviour in preschool children: The CORALS cohort

**DOI:** 10.1111/mcn.13672

**Published:** 2024-06-09

**Authors:** Ana Daniela Ortega‐Ramírez, Ivie Reis Maneschy, María L. Miguel‐Berges, Belen Pastor‐Villaescusa, Rosaura Leis, Nancy Babio, Santiago Navas‐Carretero, Olga Portoles, Ana Moreira, José Manuel Jurado‐Castro, Katherine Flores‐Rojas, Rocío Vázquez‐Cobela, Rosaura Picáns‐Leis, Gisela Mimbreros, Paloma Flores‐Barrantes, José Alfredo Martínez, Cristina Castro‐Collado, Natalia Ferré‐Pallás, Natalia Gimenez‐Legarre, Mercedes Gil‐Campos, Jordi Salas‐Salvadó, Pilar de Miguel‐Etayo, Luis A. Moreno Aznar, The Childhood Obesity Risk Assessment Longitudinal Study (CORALS)

**Affiliations:** ^1^ Growth, Exercise, Nutrition and Development (GENUD) Research Group University of Zaragoza Zaragoza Spain; ^2^ Instituto Agroalimentario de Aragón (IA2) Zaragoza Spain; ^3^ Faculty of Medicine University of Colima Colima Mexico; ^4^ Consorcio CIBER, M. P. Fisiopatología de la Obesidad y Nutrición (CIBEROBN) Instituto de Salud Carlos III (ISCIII) Madrid Spain; ^5^ Primary Care Interventions to Prevent Maternal and Child Chronic Diseases of Perinatal and Developmental Origin (RICORS), RD21/0012/0008 Instituto de Salud Carlos III Madrid Spain; ^6^ Metabolism and Investigation Unit, Reina Sofia University Hospital, Maimónides Institute of Biomedicine Research of Córdoba (IMIBIC) University of Córdoba Córdoba Spain; ^7^ Unit of Pediatric Gastroenterology, Hepatology and Nutrition, Pediatric Service Hospital Clínico Universitario de Santiago Santiago de Compostela Spain; ^8^ Pediatric Nutrition Research Group, Health Research Institute of Santiago de Compostela (IDIS), Unit of Investigation in Nutrition Growth and Human Development of Galicia‐USC Santiago de Compostela Spain; ^9^ Universitat Rovira i Virgili Departament de Bioquímica i Biotecnologia, Unitat de Nutrició Humana, Grup ANUT‐DSM Reus Spain; ^10^ Institut d'Investigació Sanitària Pere Virgili (IISPV) Reus Spain; ^11^ Center for Nutrition Research University of Navarra Pamplona Spain; ^12^ Department of Nutrition, Food Sciences and Physiology, Faculty of Pharmacy & Nutrition University of Navarra Pamplona Spain; ^13^ IdisNA, Navarra Institute for Health Research Pamplona Spain; ^14^ Department of Preventive Medicine and Public Health University of Valencia Valencia Spain; ^15^ Hospital del Mar Medical Research Institute (IMIM) Barcelona Spain; ^16^ Universitat Rovira i Virgili Departament de Medicina i Cirurgia, Unitat de Recerca en Pediatria, Nutrició i Desenvolupament Humà Tarragona Spain; ^17^ Department Physiatry and Nursing Universidad de Zaragoza Zaragoza Spain

**Keywords:** appetite, breastfeeding, childhood, complementary feeding, eating behaviour, feeding methods, infant

## Abstract

This study aimed to investigate if the duration of breastfeeding and the method at initiation of complementary feeding affect eating behaviour in children aged 3−6 years. This is a cross‐sectional analysis from the Childhood Obesity Risk Assessment Longitudinal Study project, an ongoing longitudinal cohort study that aims to identify childhood obesity risk factors in Spanish children. A total of 1215 children aged 3−6 years were included. Breastfeeding duration and the method of initiation of complementary feeding [baby‐led weaning (BLW), traditional/spoon or mixed method] were evaluated. Eating behaviour at 3−6 years was assessed with the Child Eating Behaviour Questionnaire. Generalized linear models were fitted to assess the association between the aforementioned exposures and eating behaviour.

Children breastfed for ≥4 months were less likely to be fussy eaters at 3−6 years compared to those breastfed for <1 month (OR: 0.86 95% CI: 0.76−0.98; *p* = 0.031). Compared to those children using the traditional/spoon‐feeding method, those initiating complementary feeding through BLW or through a mixed approach were more likely to have higher scores on the enjoyment of food (EF) (OR, 95% CI: 1.33, 1.13−1.57; *p* = 0.001 and 1.17, 1.05−1.30; *p* = 0.002, respectively) and lower scores on food fussiness (FF) at 3−6 years (0.76, 0.62−0.91; *p* = 0.004 and 0.87, 0.78−0.98; *p* = 0.033, respectively). Breastfeeding for ≥4 months and initiation of complementary feeding with the BLW and a mixed approach were associated with greater EF and lower FF, which should endure practice.

## Key messages

- Infants breastfed for ≥4 months have higher enjoyment of food at 3−6 years of age than those breastfed for <1 month.
- Introducingsolids with hand‐held food pieces, either fully of partially, confers greater ejoyment of food and less food fussiness at 3−6 years of age than traditional/spoon‐feeding.
- Early feeding practices influence future eating behaviour, particularly during preschool years.

## Graphical abstract

Early feeding practices may influence preschool‐age eating behaviour. Breastfeeding for ≥4 months showed increased enjoyment of food (EF) at ages 3−6 compared to <1 month. Introducing solid foods through baby‐led weaning or mixed methods was associated with higher EF and less food fussiness at 3−6 years than traditional spoon‐feeding.

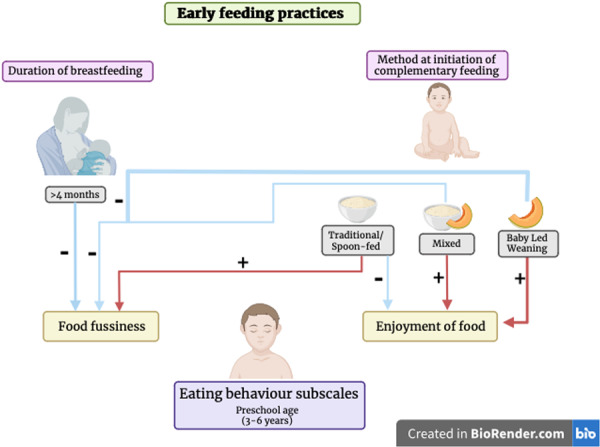

## 1 INTRODUCTION

Eating behaviour is defined as the attitudes related to the selection and decision of which foods to eat (Freitas et al., 2018), including aspects such as food enjoyment, emotional eating, response to food and satiety and food fussiness (FF) (Wardle et al., 2001). Eating behaviour is influenced by biological, environmental and social factors (Jimeno‐Martínez et al., 2021) and has been associated with the risk of childhood overweight and obesity (Carnell & Wardle, 2008; Kininmonth et al., 2021; Olwi et al., 2023). The development of eating behaviours has its origins in utero (Freitas et al., 2018). However, the first postnatal months are considered as a sensitive period for the development of healthy eating habits since biological plasticity and behavioural modelling occur (Maier‐Nöth, 2023; Ramirez‐Silva et al., 2021), including the programming of appetite regulation (Ross & Desai, 2014). Besides the intrinsic factors that influence eating behaviours, environmental factors such as early feeding practices can also shape the eating behaviours (De Cosmi et al., 2017; Jimeno‐Martínez et al., 2021; Llewellyn & Fildes, 2017) and determine long‐term eating habits, growth outcomes and future metabolic responses (Ramirez‐Silva et al., 2021).

Breastfeeding has been related to appetite regulation through different pathways, one of them is throughout flavour learning, since a variety of tastes and flavour volatiles ingested by the mother pass through breast milk (Nicklaus, 2017; Pang et al., 2020; Ventura, 2017), allowing the infant the acquisition of a taste for a variety of food (Maier‐Nöth, 2023; Mennella et al., 2001). Besides of its nutritional and immunological content, breast milk contains adipokines such as leptin and adiponectin that participate in the regulation of appetite (Ramirez‐Silva et al., 2021). Additionally, breastfeeding has been associated with a more responsive and less controlling feeding style since it allows the infant to have more control over the volume of milk and speed in every feed, unlike formula‐fed infants, where bottle‐feeding may potentially override the child's ability to self‐regulate since the caregiver has more control over the feeds (Freitas et al., 2018; Pang et al., 2020). Moreover, breastfeeding mothers may be more vigilant for the satiety and hunger signals of their infants (Masztalerz‐Kozubek et al., 2022). This self‐regulation learned by the infant through breastfeeding can continue in the complementary feeding period, which is also considered a window of opportunity for the development of eating behaviours, as the introduction of solid foods in the diet exposes the infant to a variety of new foods and sensory experiences such as textures and flavours (Boswell, 2021; De Cosmi et al., 2017; Maier‐Nöth, 2023; Nicklaus, 2017).

During the complementary feeding period, most studies have focused on the moment of introduction and the quality of food; however, as well as the timing and content of the diet, it is likely that the way in which foods are given to the infant may also influence outcomes on dietary preferences and appetite regulation (Fewtrell et al., 2017). Recently, interest in the baby‐led weaning method (BLW) has grown substantially (D'Auria et al., 2018; Masztalerz‐Kozubek et al., 2022). BLW is a responsive complementary feeding method where the infant feeds himself hand‐held foods instead of being spoon‐fed by an adult, sharing family foods and mealtimes, with the aim to allow the infant to self‐feed, thereby not imposing on how much the infant consumes (Boswell, 2021; D'Auria et al., 2018; Fewtrell et al., 2017). Even though the link between early feeding practices and eating behaviours is still inconclusive (Masztalerz‐Kozubek et al., 2022).

Therefore, the present study aimed to identify whether there is an association between some early feeding practices, such as duration of breastfeeding and the method at initiation of complementary feeding with subsequent eating behaviour in children aged 3−6 years.

## 2 METHODS

### 2.1 Study design

This is a cross‐sectional study based on the baseline data of the Childhood Obesity Risk Assessment Longitudinal Study (CORALS), a prospective ongoing multicentre cohort study in preschool children aged 3−6 years across seven Spanish cities (Barcelona, Cordoba, Pamplona, Reus, Santiago de Compostela, Valencia and Zaragoza). The main aim of the CORALS project is to identify potential risk factors for childhood obesity, and it has a 10‐year expected follow‐up (https://corals.es/). To be enroled in the study, parents or caregivers that agreed to participate in the study had to sign a consent form, attend the inclusion visit and complete several questionnaires. The exclusion criteria include belonging to a family with difficulty participating, comprehension or language difficulties and unstable residence.

For this analysis, children recruited between March 2019 and June 2021 from the CORALS study were selected. Participants who attended the baseline visit and completed the provided questionnaires, as well as those who had available data of the children eating behaviour questionnaire (CEBQ), the duration of breastfeeding, complementary feeding initiation method, birthweight for gestational age, maternal education, maternal age and maternal smoking during pregnancy were included in the present analyses. Children born premature (<37 gestational weeks) or with missing data in the variables of interest were excluded.

### 2.2 Data collection

#### 2.2.1 Parental questionnaire

A set of self‐administered questionnaires completed at home by parents or caregivers of children aged between 3 and 6 years were used to collect information on early life factors including child's sex, birthweight for gestational age, maternal data like maternal age in years, maternal education categorized according to the years of education as basic (<7−12 years), middle (13−16 years) and high (>16 years), maternal smoking during pregnancy and lifestyle patterns, among others. Early feeding practices like breastfeeding (months), age at initiation of solid foods, age at introduction of formula and complementary feeding initiation method were recorded retrospectively. Breastfeeding duration was classified as <1, 1−3 and ≥4 months. The complementary feeding method at the initiation of the introduction of solids was classified as traditional/spoon‐feeding (mashed food or porridge, fed by an adult), mixed method (approximately 50% mashed food and 50% hand‐held size pieces of food) and BLW defined as baby‐fist or hand‐held size pieces of food, infant eating autonomously. BLW is a responsive complementary feeding method promotes an active role of the infant in the feeding process, thus at 6 months of age, the infant feeds himself ‘whole’ pieces of food sharing family foods and mealtimes (Bocquet et al., 2022; D'Auria et al., 2018; Fewtrell et al., 2017).

#### 2.2.2 CEBQ

Eating behaviour was assessed with the CEBQ. The CEBQ is a parent report questionnaire developed in the United Kingdom (Wardle et al., 2001) and has been validated across different countries, including Spain (Jimeno‐Martínez et al., 2022). It classifies eating behaviour into two domains: food approach and food avoidance, each domain is composed of four appetitive traits or scales. Enjoyment of Food (EF), Food Responsiveness (FR), Desire to Drink (DD) and Emotional Overeating (EOE) correspond to the food approach domain, while Satiety Responsiveness (SR), Slowness in Eating (SE), FF and Emotional Undereating (EUE) are considered food avoidance scales. The CEBQ is composed of 35 items answered by parents/caregivers in a 5‐point Likert‐type scale, where 1 is complete absence (never) and 5 is the highest intensity of specific eating behaviour (always). Mean scores for each subscale were calculated and used for the analysis.

### 2.3 Statistical analysis

All statistical analyses were performed using the Statistical Package for Social Sciences version 25.0 (SPSS Inc.). The data are presented as means ± SD for quantitative variables and as percentages for qualitative variables. To compare the eating behaviour subscales (dependent variables/outcome) among breastfeeding duration and complementary feeding method categories (independent variables/exposures) with the adjustment of cofounders (birthweight for gestational age, child's gender, maternal age, maternal education, maternal smoking during pregnancy), ANCOVA test was conducted. Generalized linear models were used to assess the associations between breastfeeding duration and complementary feeding method categories and the eating behaviour subscales and expressed as odds ratios (OR) with 95% confidence intervals (CI). *p* Value lower than 0.05 was considered as statistically significant.

### 2.4 Ethics Statement

Before the enrolment of participants in the Corals study, approval for the protocol was secured from the ethics committees of all seven participating cities (reference nos. 051/2019, 4155/2019, 2019/18, 9/19, 09/2019, 19/27 and 2019/131). All questionnaires were completed by parents/legal guardians who gave written informed consent, and the statements of the Declaration of Helsinki were followed.

## 3 RESULTS

**Figure 1:**
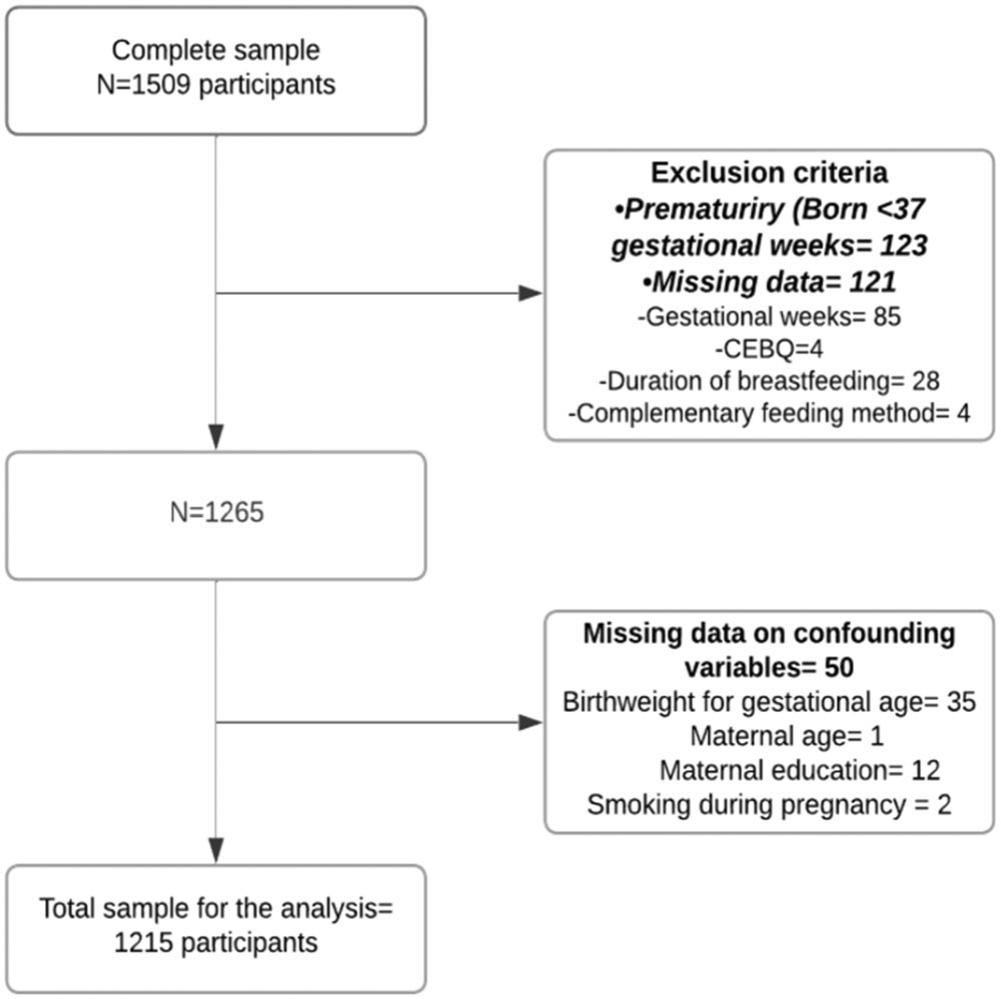
Study flowchart of the participants from the CORALS study. CEBQ, child eating behaviour questionnaire; CORALS, Childhood Obesity Risk Assessment Longitudinal Study.

From the total sample (*n* = 1509), 208 children did not meet the inclusion criteria or had missing data on gestational weeks at birth, 36 had missing data on CEBQ, duration of breastfeeding or complementary feeding method, while 50 had missing data on the confounding variables (birthweight for gestational age, maternal education, maternal age and smoking during pregnancy). Thus, a final sample of 1215 children were included in this analysis (Figure 1).

Table 1 shows the general characteristics of the sample. Mean age of children at the inclusion in the study was 4.7 ± 1.0 years, 50.4% were girls, 79.3% were born with an appropriate birthweight for gestational age (AGA), and 76.4% were delivered by vaginal birth. Most of the children were breastfed for ≥4 months (63.4%) and initiated complementary feeding with the traditional/spoon‐feeding method (69.7%). The mean age at the introduction of solid foods and for the introduction of formula was 5.9 ± 2.3 and 3.1 ± 3.6 months, respectively.

**Table 1:** Descriptive characteristics of the sample.

Variable	*N* (%) or mean ± SD
Child's sex	
Boy	603 (49.6)
Girl	612 (50.4)
Child age at inclusion in the study (years)	4.7 ± 1.0
Birthweight for gestational age	
Small for gestational age	106 (8.7)
Appropriate for gestational age	963 (79.3)
Large for gestational age	106 (12.0)
Gestational age (weeks)	39.5 ± 1.3
Mode of delivery	
Vaginal	938 (76.4)
Caesarean section	290 (23.6)
Gestational weight gain (kg)	12.4 ± 5.8
Maternal smoking during pregnancy	
Yes	149 (12.3)
No	1066 (87.7)
Maternal age at childbirth (years)	33.0 ± 4.8
Maternal education	
Basic	210 (17.3)
Middle	379 (31.2)
High	626 (51.5)
Breastfeeding (month)	
<1	238 (19.6)
1−3	207 (17.0)
≥4	770 (63.4)
Breastfeeding duration (months)	7.8 ± 9.4
Age of introduction of formula (months) *N* = 924	3.1 ± 3.6
Age at introduction of solid foods (months) *N* = 895	5.9 ± 2.3
Complementary feeding method	
Traditional/spoon‐feeding	847 (69.7)
Mixed	275 (22.6)
Baby‐led weaning (BLW)	93 (7.7)
Children's eating behaviour questionnaire subscales (score)	
Enjoyment of food	3.38 ± 0.77
Food responsiveness	2.14 ± 0.86
Desire to drink	2.27 ± 0.90
Emotional overeating	1.66 ± 0.59
Food fussiness	2.90 ± 0.88
Satiety responsiveness	2.78 ± 0.71
Slowness in eating	2.91 ± 0.84
Emotional undereating	2.65 ± 0.91

**Figure 2:**
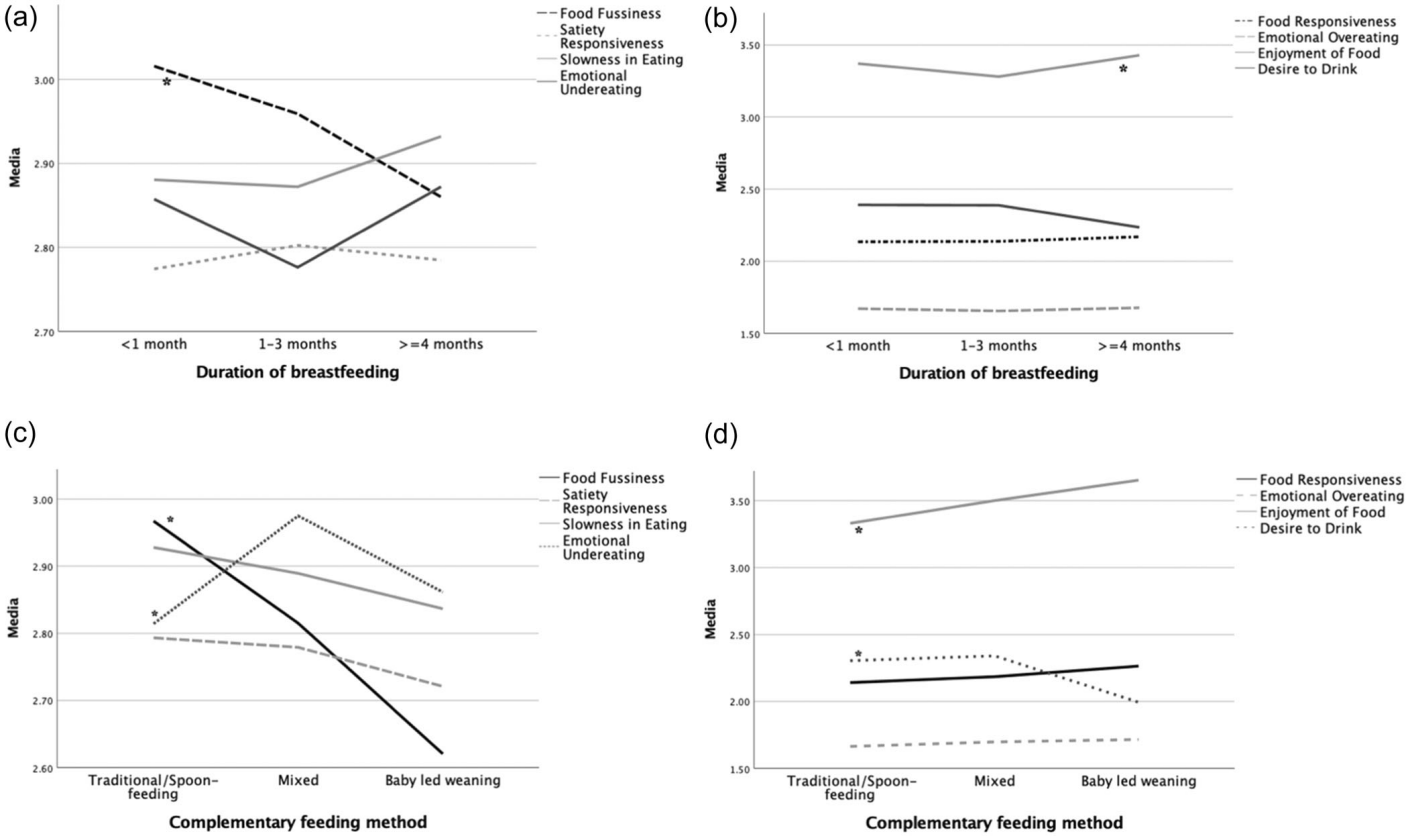
Mean scores for CEBQ scales according to breastfeeding duration and complementary feeding method categories: (a) Food avoidance subscales for the duration of breastfeeding. (b) Food approach subscales for the duration of breastfeeding. (c) Food avoidance subscales for complementary feeding methods. (d) Food approach subscales for complementary feeding methods. *ANOVA test; significant differences (*p* < 0.05) between EBF <1 versus >4 months for food fussiness (FF) and desire to drink (DD), and EBF for 1−3 versus >4 months for enjoyment of food (EF). Significant differences (*p* < 0.05) between the traditional/spoon‐feeding method versus BLW and mixed feeding for FF and EF, traditional/spoon‐feeding versus BLW for DD and versus mixed feeding for emotional undereating. BLW, baby‐led weaning; CEBQ, child eating behaviour questionnaire; EBF, exclusive breastfeeding.

**Figure 3:**
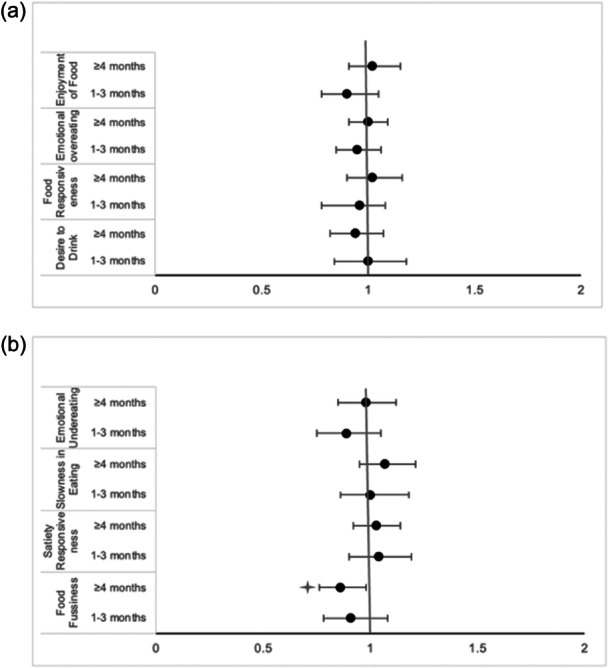
Odds ratios (95% CI) for CEBQ subscales according to breastfeeding categories: (a) Food approach subscales. (b) Food avoidance subscales. *Ref. <1 month. Adjusted by child's sex, birthweight for gestational age, maternal age and education. CEBQ, child eating behaviour questionnaire.

The mean scores of the CEBQ subscales according to the duration of breastfeeding and the complementary feeding groups can be seen in Tables S1 and S2, respectively. Results of OR for each CEBQ subscale according to breastfeeding duration unadjusted and adjusted by confounding variables are showed in Table S3. When comparing breastfeeding ≥4 versus <1 month, without the adjustment for covariables, a statistically significant difference was found in FF (*p* = 0.016) and in DD (*p* = 0.028). Figure 2 illustrates these differences, noting a decrease in FF and DD according to the months of breastfeeding, while the EF shows an increase from <1 month of breastfeeding to ≥ 4 months. Figure 3 shows the relationship between the duration of breastfeeding and the CEBQ adjusted for cofounding variables sex, birthweight for gestational age, maternal age and education. Only FF was found to be statistically significant, breastfeeding for ≥4 months was associated with a lower mean score on this trait at 3−6 years, compared to those breastfed for <1 month (OR: 0.86, 95% CI: 0.76−0.98; *p* = 0.031). There was no association between the duration of breastfeeding and the other eating behaviour subscales.

**Figure 4:**
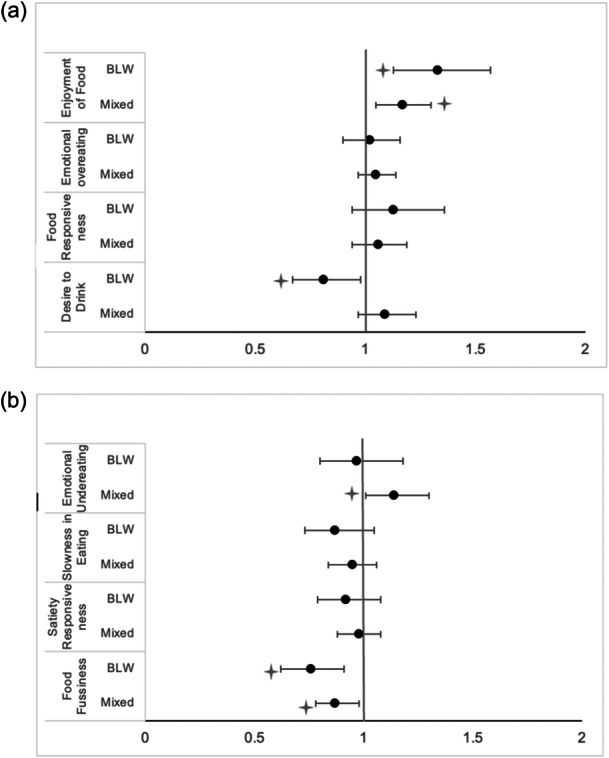
Odds ratios (95% CI) for CEBQ subscales according to complementary feeding methods: (a) Food approach subscales. (b) Food avoidance subscales. *Ref, Traditional/spoon‐fed. Adjusted by child's sex, birthweight for gestational age, maternal age and education and duration of breastfeeding. CEBQ, child eating behaviour questionnaire.

In the comparison between the complementary feeding groups and the eating behaviour subscales, without covariates, significant trends for FF, EF, DD and EUE were observed (*p* < 0.05) (Figure 2). When adjusting for cofounding variables, a significant difference in the EF and FF between the three groups was found, those who started solids with the BLW approach and in a mixed way had higher scores on EF (OR: 1.33 95% CI: 1.13−1.57, *p* = 0.001 and OR: 1.17 95% CI: 1.05−1.30, *p* = 0.002, respectively) and lower scores on FF (OR: 0.76 95% CI: 0.62−0.91, *p* = 0.004 and OR: 0.87 95% CI: 0.78−0.98, *p* = 0.033, respectively) compared to the traditional/spoon‐feeding group. While a significant difference was also found between the BLW group and the traditional/spoon‐feeding group for DD, and between the mixed group and the traditional/spoon‐feeding group for EUE; the BLW group had lower DD (OR: 0.81 95% CI: 0.67−0.98, *p* = 0.032) and the mixed group had higher EUE (OR: 1.14 95% CI: 1.01−1.30, *p* = 0.030) at 3−6 years than children in the traditional/spoon‐feeding group (Figure 4 and Table S4).

## 4 DISCUSSION

The main results of this study are in line with the findings in previous studies (Cox et al., 2024; Fu et al., 2018; Komninou et al., 2019; Pang et al., 2020; Specht et al., 2018; Taylor & Emmett, 2019): longer duration of breastfeeding, as well as the choice of a responsive feeding method like BLW or the mixed method are associated with a lower FF at the age of 3−6 years.

### 4.1 Duration of breastfeeding and eating behaviour

In the present study, only the relationship between duration of breastfeeding and FF was found; those infants who were breastfed for ≥4 months had lower FF at 3−6 years of age than those who were breastfed for <1 month. Similarly, other authors found that children who received exclusive breastfeeding (EBF) for 4−5 months or who had a high breastfeeding exposure (EBF for 4 months and continued to breastfeed at least partially until at least 6 months) were less picky with foods and had a lower FF at preschool age than those who had EBF < 1 month or with a low breastfeeding exposure (weaned from breast milk or exclusively formula‐fed before the age of 3 months) (Pang et al., 2020; Specht et al., 2018). Also, another study found that infants who were breastfed for less than 2 months had a higher FF score at 4 years old than those who were breastfed for 6 months or more (Taylor & Emmett, 2019). According to Ventura et al., infants' food preferences may emerge as a result of three mechanisms: repeated exposure, variety exposure and associative conditioning (Ventura, 2017). Breastfeeding favours the acquisition of a taste for a variety of foods through repeated exposure to different flavours (Forestell, 2016; Maier‐Nöth, 2023; Nicklaus, 2017), as well as exposure to variations in nutrient and flavour composition across feeding episodes that reflect the maternal diet. Therefore, breastfed infants experience the flavours of their mother's diet before they experience them in solid foods (Mennella et al., 2001; Nicklaus, 2017). This may be reflected in greater acceptance and intake of a variety of foods, such as vegetables, in childhood (Maier‐Nöth et al., 2016; Möller et al., 2013; Specht et al., 2018). Additionally, breastfed infants exhibit lower levels of neophobia and picky eating compared to formula‐fed children (Specht et al., 2018; Ventura, 2017). Thus, early development of taste and food pleasure has long‐lasting influences on subsequent food preferences and choices (Maier‐Nöth, 2023).

Some studies have found that longer duration of breastfeeding is positively associated with other food avoidance subscales such as SR and SE, and negatively associated with food approach subscales: FR, EOE, EF and DD (Brown & Lee, 2012; Magdy Omar et al., 2022; Masztalerz‐Kozubek et al., 2022; Yelverton et al., 2021). However, we did not find an association between the duration of breastfeeding and these eating behaviour subscales.

### 4.2 Method at initiation of complementary feeding and eating behaviour

This study found that those who started complementary feeding with the BLW method had higher scores in EF and lower scores in FF and DD at 3−6 years of age compared to those who started with the traditional/spoon‐feeding method. Similarly, starting with a mixed method (50% spoon‐fed foods and 50% hand‐held size pieces of food) was associated with higher EF and lower FF than the traditional method but lower EF and higher FF than those who started with the BLW method. This is in agreement with the results of Fu et al. (2018), Komninou et al. (2019) and Cox et al. (2024), showing that infants fed with the BLW exhibited lower FF scores compared to those fed by the traditional method during the first years of life, while the study conducted by Masztalerz‐Kozubek et al. (2022) found also higher EF scores among infants fed by the BLW. Additionally, Taylor et al. (2017) reported that introducing solids using the BLISS method was associated with decreased FF and increased EF at 12 months.

Picky or fussy eating is a complex behaviour characterized by food selectivity, sensory sensitivity and lack of interest in eating (Samuel et al., 2018). In general, picky eating behaviours are common across preschool children (Van Der Horst et al., 2016). Parental feeding styles, reduced duration of EBF, late introduction of complementary feeding as well as late introduction of chewy foods have been related to the development of picky eating (Taylor et al., 2015).

Infants fed with the BLW approach are thought to accept a wider range of foods because of the exposure to different food tastes and textures (D'Auria et al., 2018). Infants who are not exposed to different textures earlier and more often during complementary feeding are more likely to reject foods by their texture, later in life (Bocquet et al., 2022; Cox et al., 2024). Responsive feeding characterized by an awareness of hunger and satiety cues is linked to reduced levels of fussy eating (Wolstenholme et al., 2020). The responsive approach of BLW is related to positive parental practices like less pressure to eat, less use of food as a reward, and those infants are often involved in sharing family foods and mealtimes; this may lead to less difficult feeding and healthier eating behaviours (Bocquet et al., 2022; Cox et al., 2024; D'Auria et al., 2018). Therefore, introducing complementary foods with more complex textures accompanied by a responsive feeding approach may foster a positive relationship with food and enhance mealtime enjoyment, explaining the higher EF and the less FF scores in the BLW group in preschoolers.

Nevertheless, it is important to highlight that BLW infants may consume family foods, which may not always be suitable for infants (Daniels et al., 2015; D'Auria et al., 2018). Concerns have been expressed that infants following the BLW strategy might be exposed to sugar and salt tastes, potentially resulting in their increased consumption (D'Auria et al., 2018), which may also be related to a higher EF. However, EF has shown to be related to a greater consumption of healthy foods in children and adolescents (Carnell et al., 2016; Jalkanen et al., 2017).

The complementary feeding approach chosen may influence an infant's food choices and the relationship towards food for the rest of the infant's life (Bocquet et al., 2022). One of the main benefits that the BLW method advocates is an infant's appetite control by providing early and more stable learning about the satiating capacities of foods, and some studies have linked a greater SR with the BLW method (D'Auria et al., 2018; Martinón‐Torres et al., 2021) However, the results are inconsistent (Brown & Lee, 2013; Cameron et al., 2015; Daniels et al., 2015). Notably, our study found no association between SR and the method at the introduction of complementary feeding.

The recommendation on the best method for initiating complementary feeding is not yet defined (Fewtrell et al., 2017). However, both the World Health Organization and The European Society for Paediatric Gastroenterology, Hepatology and Nutrition (ESPGHAN) emphasize the importance of ensuring that complementary feeding is safe and offers an adequate variety of foods, appropriate frequency and portion sizes and foods with texture and consistencies suitable for the infant's developmental stage. In addition, parents should be encouraged to adopt a responsive style of parenting and understand how to recognize their infant's hunger and satiety cues (Fewtrell et al., 2016; Hetherington et al., 2011; Organización Mundial de la Salud, 2010).

Nonetheless, the results of this study propose that early feeding practices such as longer duration of breastfeeding and initiating complementary feeding with a responsive feeding method and early introduction of textures like the BLW method or even in a mixed way (50% puree foods and 50% hand‐held size pieces of food) could potentially influence on the development of healthy eating behaviours at preschool age, through less FF and greater EF. These practices may contribute to preventing picky/fussy eating and fostering a positive relationship with food.

### 4.3 Limitations and strengths

Some limitations of this study deserve to be mentioned. First, not all the feeding variables in the first months of life were evaluated. Second, the duration of breastfeeding and the method at initiating complementary feeding were retrospectively reported by parents through a self‐reported questionnaire, so a recall bias would be possible; this prevented the classification of breastfeeding as either exclusive or partial and to determine if there were any differences in eating behaviour between the two; also this also led to a more general definition of complementary feeding methods than other author's definitions. Third, the majority of the mothers of the participants, presented middle to high level of education, therefore the results may not be representative for all the educational levels. Among the strengths of this study, this is a larger sample than the ones included in other studies, while the Spanish version of the CEBQ that was used has been validated previously with a high reliability and internal validity, making it a useful tool for the assessment of eating behaviour in this population.

## 5 CONCLUSION

Breastfeeding for 4 months or more and initiating complementary feeding with the BLW method or in a mixed way (50% spoon‐fed and 50% hand‐held size pieces of food) were associated with a better relationship towards food at 3−6 years of age, through greater EF and lower FF. The association between EF and FF with the method of initiation of complementary feeding is gradual. Infants who initiated with the BLW method tended to exhibit higher levels of EF and lower of FF compared to those who began with the mixed method. Similarly, those who initiate with the mixed method tend to display higher EF and lower FF compared to those infants introduced with the traditional spoon‐feeding method. These findings contribute to the body of evidence on how early and responsive feeding practices can positively influence future eating behaviour in children. Future research should explore the impact of these feeding practices over extended periods, including the influence of parental styles and on different populations, as well as their impact on further health outcomes.

## AUTHOR CONTRIBUTIONS

Ana Daniela Ortega‐Ramírez participated in the conceptualization, formal analysis, visualization and writing of the original draft. Ivie Reis Maneschy and María L. Miguel‐Berges contributed to the investigation, formal analysis and writing of the original draft. Luis A. Moreno Aznar conducted the conceptualization, methodology, formal analysis, visualization, writing—review and editing of the manuscript. Belen Pastor‐Villaescusa, Rosaura Leis, Nancy Babio, Santiago Navas‐Carretero, Olga Portoles, Ana Moreira, José Manuel Jurado‐Castro, Katherine Flores‐Rojas, Rocío Vázquez‐Cobela, Gisela Mimbreros, Paloma Flores‐Barrantes, José Alfredo Martínez, Cristina Castro‐Collado, Natalia Ferré‐Pallás, Natalia Gimenez‐Legarre, Mercedes Gil‐Campos, Jordi Salas‐Salvadó and Pilar de Miguel‐Etayo participated in writing—review and editing of the manuscript. All authors have revised and approved the final version of the manuscript.

## CONFLICT OF INTEREST STATEMENT

The authors declare no conflict of interest.

## Supporting information

Supporting information.

Supporting information.

Supporting information.

Supporting information.

## Data Availability

The data that support the findings of this study are available on request from the corresponding author. The data are not publicly available due to privacy or ethical restrictions.
