# Supplementary material for: Early feeding practices and eating behaviour in preschool children: The CORALS cohort
Source: Matern Child Nutr. 2024 Jun 9;20(4):e13672. doi: 10.1111/mcn.13672 (PMC11574645; doi:10.1111/mcn.13672)
Supplement: Supplementary file 1 — Supporting information. [file MCN-20-e13672-s003.docx]

**Table S1. Mean scores of the CEBQ subscales according with the duration of breastfeeding.**

| CEBQ subscales | <1 month | 1-3 months | ≥ 4 months | p value |
| --- | --- | --- | --- | --- |
| Food fussiness | 3.0 ± 0.8 | 2.9 ± 0.8 | 2.8 ± 0.8 | **0.032** |
| Food responsiveness | 2.1 ± 0.8 | 2.1 ± 0.9 | 2.1 ± 0.8 | 0.808 |
| Emotional overeating | 1.6 ± 0.6 | 1.6 ± 0.5 | 1.6 ± 0.6 | 0.893 |
| Enjoyment of food | 3.3 ± 0.7 | 3.2 ± 0.8 | 3.4 ± 0.7 | **0.039** |
| Desire to drink | 2.3 ± 0.9 | 2.3 ± 1.0 | 2.2 ± 0.8 | **0.014** |
| Satiety responsiveness | 2.7 ± 0.7 | 2.8 ± 0.7 | 2.7 ± 0.7 | 0.915 |
| Slowness in eating | 2.8 ± 0.8 | 2.8 ± 0.8 | 2.9 ± 0.8 | 0.525 |
| Emotional undereating | 2.8 ± 0.9 | 2.7 ± 0.8 | 2.8 ± 0.9 | 0.395 |

Data presented as mean ± SD. CEBQ: Child Eating Behaviour Questionnaire.
