# Supplementary material for: Early feeding practices and eating behaviour in preschool children: The CORALS cohort
Source: Matern Child Nutr. 2024 Jun 9;20(4):e13672. doi: 10.1111/mcn.13672 (PMC11574645; doi:10.1111/mcn.13672)
Supplement: Supplementary file 2 — Supporting information. [file MCN-20-e13672-s001.docx]

**Table S2. Mean scores of the CEBQ subscales according with the complementary feeding method.**

| CEBQ subscales | Traditional/ Spoon-fed | Mixed | BLW | p |
| --- | --- | --- | --- | --- |
| Food fussiness | 2.9 ± 0.8 | 2.8 ± 0.8 | 2.6 ± 0.7 | **<0.001** |
| Food responsiveness | 2.1 ± 0.8 | 2.1 ± 0.8 | 2.2 ± 0.8 | 0.352 |
| Emotional overeating | 1.6 ± 0.5 | 1.6 ± 0.6 | 1.7 ± 0.6 | 0.565 |
| Enjoyment of food | 3.3 ± 0.7 | 3.5 ± 0.7 | 3.6 ± 0.7 | **<0.001** |
| Desire to drink | 2.3 ± 0.9 | 2.3 ± 0.9 | 1.9 ± 0.7 | **0.003** |
| Satiety responsiveness | 2.7 ± 0.7 | 2.7 ± 0.6 | 2.7 ± 0.6 | 0.630 |
| Slowness in eating | 2.9 ± 0.8 | 2.8 ± 0.8 | 2.8 ± 0.7 | 0.512 |
| Emotional undereating | 2.8 ± 0.9 | 2.9 ± 0.9 | 2.8 ± 0.9 | **0.037** |

Data presented as mean ± SD. CEBQ: Child Eating Behaviour Questionnaire. BLW: Baby led weaning.
