# Supplementary material for: Early feeding practices and eating behaviour in preschool children: The CORALS cohort
Source: Matern Child Nutr. 2024 Jun 9;20(4):e13672. doi: 10.1111/mcn.13672 (PMC11574645; doi:10.1111/mcn.13672)
Supplement: Supplementary file 3 — Supporting information. [file MCN-20-e13672-s002.docx]

**Table S3. Multivariable model for CEBQ scales according with the duration of breastfeeding.**

| CEBQ scales | Bivariate model | | Multivariable model* | | |  |
| --- | --- | --- | --- | --- | --- | --- |
|  | **OR (IC 95%)** | **p** | | **OR (IC 95%)** | **p** | |
| Food fussiness |  |  | |  |  | |
| <1 month  1-3 months  ≥4 months | Ref  0.93 (0.79-1.10)  0.85 (0.75-0.97) | 0.454  **0.016** | | Ref  0.91 (0.78-1.08)  0.86 (0.76-0.98) | 0.310  **0.031** | |
| Food responsiveness |  |  | |  |  | |
| <1 month  1-3 months  ≥4 months | Ref  0.97 (0.82-1.14)  1.02 (0.90-1.15) | 0.761  0.737 | | Ref  0.96 (0.82-1.13)  1.02 (0.90-1.16) | 0.687  0.665 | |
| Emotional overeating |  |  | |  |  | |
| <1 month  1-3 months  ≥4 months | Ref  0.94 (0.84-1.05)  0.99 (0.91-1.08) | 0.347  0.877 | | Ref  0.95 (0.85-1.06)  1.00 (0.91-1.09) | 0.393  0.946 | |
| Enjoyment of food |  |  | |  |  | |
| <1 month  1-3 months  ≥4 months | Ref  0.90 (0.78-1.04)  1.04 (0.93-1.16) | 0.177  0.469 | | Ref  0.90 (0.78-1.05)  1.02 (0.91-1.15) | 0.192  0.672 | |
| Desire to drink |  |  | |  |  | |
| <1 month  1-3 months  ≥4 months | Ref  0.99 (0.84-1.17)  0.86 (0.75-0.98) | 0.950  **0.028** | | Ref  1.00 (0.84-1.18)  0.94 (0.82-1.07) | 0.998  0.383 | |
| Satiety responsiveness |  |  | |  |  | |
| <1 month  1-3 months  ≥4 months | Ref  1.03 (0.90-1.18)  1.01 (0.91-1.12) | 0.578  0.774 | | Ref  1.04 (0.90-1.19)  1.03 (0.92-1.14) | 0.563  0.579 | |
| Slowness in eating |  |  | |  |  | |
| <1 month  1-3 months  ≥4 months | Ref  1.00 (0.86-1.17)  1.06 (0.94-1.19) | 0.931  0.337 | | Ref  1.00 (0.86-1.18)  1.07 (0.95-1.21) | 0.908  0.249 | |
| Emotional undereating |  |  | |  |  | |
| <1 month  1-3 months  ≥4 months | Ref  0.90 (0.76-1.07)  1.00 (0.88-1.14) | 0.264  0.933 | | Ref  0.89 (0.75-1.05)  0.98 (0.85-1.12) | 0.185  0.807 | |

CEBQ: Child Eating Behaviour Questionnaire.

*Multivariate model adjusted for sex, maternal age, maternal education, and birthweight for gestational age.
