# Supplementary material for: Early feeding practices and eating behaviour in preschool children: The CORALS cohort
Source: Matern Child Nutr. 2024 Jun 9;20(4):e13672. doi: 10.1111/mcn.13672 (PMC11574645; doi:10.1111/mcn.13672)
Supplement: Supplementary file 4 — Supporting information. [file MCN-20-e13672-s004.docx]

**Table S4. Multivariable model for CEBQ scales according with the complementary feeding method.**

| CEBQ scales | Bivariate model | | | Multivariable model* | |
| --- | --- | --- | --- | --- | --- |
|  | **OR (IC 95%)** | **p** | **OR (IC 95%)** | | **p** |
| Food fussiness |  |  |  | |  |
| Traditional/Spoon-fed  Mixed  BLW | Ref  0.86 (0.76-0.97)  0.72 (0.60-0.87) | **0.017**  **0.001** | Ref  0.87 (0.78-0.98)  0.76 (0.62-0.91) | | **0.033**  **0.004** |
| Food responsiveness |  |  |  | |  |
| Traditional/Spoon-fed  Mixed  BLW | Ref  1.05 (0.93-1.18)  1.13 (0.94-1.36) | 0.371  0.184 | Ref  1.06 (0.94-1.19)  1.13 (0.94-1.36) | | 0.325  0.188 |
| Emotional overeating |  |  |  | |  |
| Traditional/Spoon-fed  Mixed  BLW | Ref  1.04 (0.96-1.13)  1.02 (0.90-1.16) | 0.274  0.739 | Ref  1.05 (0.97-1.14)  1.02 (0.90-1.16) | | 0.222  0.725 |
| Enjoyment of food |  |  |  | |  |
| Traditional/Spoon-fed  Mixed  BLW | Ref  1.18 (1.06-1.31)  1.36 (1.15-1.60) | **0.001**  **0.000** | Ref  1.17 (1.05-1.30)  1.33 (1.13-1.57) | | **0.002**  **0.001** |
| Desire to drink |  |  |  | |  |
| Traditional/Spoon-fed  Mixed  BLW | Ref  1.05 (0.93-1.19)  0.74 (0.61-0.90) | 0.360  **0.003** | Ref  1.09 (0.97-1.23)  0.81 (0.67-0.98) | | 0.134  **0.032** |
| Satiety responsiveness |  |  |  | |  |
| Traditional/Spoon-fed  Mixed  BLW | Ref  0.98 (0.88-1.08)  0.92 (0.79-1.08) | 0.690.0.341 | Ref  0.98 (0.88-1.08)  0.92 (0.79-1.08) | | 0.690  0.343 |
| Slowness in eating |  |  |  | |  |
| Traditional/Spoon-fed  Mixed  BLW | Ref  0.96 (0.86-1.08)  0.89 (0.75-1.07) | 0.534  0.242 | Ref  0.95 (0.84-1.06)  0.87 (0.73-1.05) | | 0.399  0.165 |
| Emotional undereating |  |  |  | |  |
| Traditional/Spoon-fed  Mixed  BLW | Ref  1.16 (1.03-1.32)  1.00 (0.82-1.22) | **0.015**  0.971 | Ref  1.14 (1.01-1.30)  0.97 (0.80-1.18) | | **0.030**  0.806 |

CEBQ: Child Eating Behaviour Questionnaire. BLW: Baby led weaning.

*Multivariate model adjusted for sex, maternal age, maternal education, and breastfeeding duration.
